# VDAC as a Cellular Hub: Docking Molecules and Interactions

**DOI:** 10.3390/ijms24076649

**Published:** 2023-04-02

**Authors:** Hanna Kmita, Angela Anna Messina, Vito De Pinto

**Affiliations:** 1 Department of Bioenergetics, Institute of Molecular Biology and Biotechnology, Faculty of Biology, Adam Mickiewicz University, Uniwersytetu Poznańskiego 6, 61-614 Poznań, Poland; 2 Molecular Biology Laboratory, Department of Biological, Geological and Environmental Sciences, University of Catania, Via S. Sofia 64, 95123 Catania, Italy; mess@unict.it; 3 Department of Biomedical and Biotechnological Sciences, University of Catania, Via S. Sofia 64, 95123 Catania, Italy; vdpbiofa@unict.it

The voltage-dependent anion channel (VDAC) is the primary regulating pathway of water-soluble metabolites and ions across the mitochondrial outer membrane. The VDAC was discovered over 45 years ago and has been extensively studied since then. It is the most abundant protein in the mitochondrial outer membrane. Accordingly, atomic force microscopy has revealed that the membrane is, in fact, peppered with pores formed by the VDAC. Thus, in addition to its transport function, the VDAC is assumed to play a structural role in the membrane.

Data suggest that physiological VDAC function is dependent on the molecular plasticity of its channel, which determines its permeability and is caused by protein interactions with numerous cellular proteins as well as molecules of endogenous and exogenous origin. The channel is produced by a single polypeptide forming a *β*-barrel. The determination of its three-dimensional structure, as well as the recognition of VDAC paralogous functional differentiation, were ground-breaking discoveries that fueled the search for VDAC interactors.

These studies tend to identify regions of the channel responsible for biological functions, owing to multiple interactions. As research and technology progress, new applications emerge as a result of the VDAC’s role as “governor” of the mitochondrion, i.e., a key regulator of mitochondrial intracellular dynamics and functions. Mitochondria are involved in a variety of cellular processes, including energy transformation, metabolism, cell proliferation, differentiation, senescence, and death. Therefore, it is not surprising that the VDAC is a rational candidate for preventing, diagnosing, or treating mitochondrial dysfunction associated with various human diseases. Importantly, there is no apparent structural, functional, or sequence similarity between natural and synthetic VDAC interactors. The lack of high specificity to the VDAC of most of the reported compounds, as well as the absence of known molecular mechanisms of action, must be overcome in order to develop VDAC-based pharmacology.

This Special Issue of the *International Journal of Molecular Sciences*, entitled “VDAC as a Cellular Hub: Docking Molecules and Interactions”, comprises a total of seven contributions, two of which are articles and five of which are reviews that present and summarize the most recent information about the VDAC’s contribution to cellular functioning based on a network of interacting factors affecting its channel activity, including its gating, selectivity, and conductance. 

From an evolutionary perspective, Mannella [1] considers VDAC properties essential for the channel’s role as a hub protein critical for the integration of mitochondria into cellular metabolic and signaling pathways. The author focuses on three relevant properties of the VDAC: remarkable structural flexibility, the ability to respond to various signals by conformational switching, and the ability to interact with different partners. 

Rostovtseva et al. [2] focus on the role of membrane-bound inhibitors with disordered polyanionic C-terminal domains in VDAC-mediated regulation of mitochondrial respiration. The inhibitors are found in abundant cytosolic proteins, tubulin and α-synuclein, which dock with the VDAC via a novel mechanism in which the transmembrane potential draws their disordered, polyanionic C-terminal domains into and through the VDAC channel. This results in reduced metabolite flux and increased calcium flux, which modulates mitochondrial potential and ATP production. The authors discuss VDAC interactions with docking proteins as well as metabolite fluxes through the VDAC that is regulated by these interactions. As a result, there is a solid foundation for discovering novel potent endogenous regulators, including VDAC inhibitors.

Pittala et al. [3] present current knowledge on post-translational modifications of VDAC paralogs, which are crucial for the protein to mediate and regulate the integration of mitochondrial functions with cellular activities. The authors focus on the new mass spectrometry techniques that are essential for the modification detection and the detected modifications in relation to human degenerative diseases.

Rister et al. [4] review roles of a glutamate residue at position 73 (E73) in VDAC regulation at multiple levels. It is proposed that the residue located on the outside of the channel and facing the hydrophobic membrane environment, is a site of multiple interactions of the VDAC with molecular partners and cellular factors. The authors indicate how the interactions and their underlying mechanisms act dependently of each other, and how a complex interplay between interacting partners and factors adapts VDAC function to the current state of the cell.

Sander et al. [5] discuss the role of the VDAC as a physiological regulator of mitochondrial Ca^2+^ uptake in the mitochondrial outer membrane. The authors focus on potential regulatory mechanisms of mitochondrial Ca^2+^ uptake through the VDAC, functioning at various levels and ranging from VDAC expression to interaction with membrane lipids and different proteins, including those forming Ca^2+^ release sites from the endoplasmic/sarcoplasmic reticulum, as well as the intrinsic properties of the VDAC, which can adapt to multiple states of conductance. The authors also review the potential roles of the mechanisms in cell physiology and pathophysiology.

The study by Shteinfer-Kuzmine et al. [6] focuses on the mechanism of misfolded SOD1-inducing cytotoxicity in ALS pathogenesis. The authors have already established that the mechanism includes misfolded SOD1 interaction with VDAC1 at its N-terminus. Consequently, different versions of the N-terminal-derived peptides inhibit the cytotoxicity of misfolded SOD1. In their present study, the VDAC1 binding site in SOD1 was identified, and two SOD1-derived peptides interacting with VDAC1 were obtained. Moreover, two VDAC1-interacting molecules that prevent VDAC1 oligomerization were obtained, and their effect on misfolded SOD1 cytotoxicity was investigated. The results indicate that SOD1-derived peptides interacting with VDAC1 and VDAC1-N-terminus-derived peptides interacting with SOD1 are potential candidates to target VDAC1-misfolded SOD1 interactions and can be regarded as important for the development of new ALS therapies. The same is true for molecules that prevent VDAC1 oligomerization, which may improve or preserve ALS patients’ daily functioning and quality of life. 

Saidani et al. [7] analyze the VDAC open state selectivity inversion. The observations made for fungi and mammalian VDAC proteins indicate that the open state of the VDAC can randomly switch between anion and cation selectivity without a change in conductance. This result calls into question the VDAC conductance–selectivity coupling dogma, but the underlying mechanism(s) remains elusive. Since VDAC proteins from different organisms share functional and structural properties, the phenomenon was investigated for a reconstituted plant VDAC. The authors prove that stigmasterol, as well as the ionic strength and magnitude of the ion concentration gradient, all influence the occurrence of selectivity inversion. Furthermore, the authors emphasize the significance of sterol–protein interactions in modulating VDAC properties.
